# *Lactobacillus reuteri* DSM 17938 and Magnesium Oxide in Children with Functional Chronic Constipation: A Double-Blind and Randomized Clinical Trial

**DOI:** 10.3390/nu12010225

**Published:** 2020-01-15

**Authors:** Megumi Kubota, Kazuya Ito, Kazuhiko Tomimoto, Mitsuharu Kanazaki, Kei Tsukiyama, Akio Kubota, Haruo Kuroki, Mitsugu Fujita, Yvan Vandenplas

**Affiliations:** 1 Kubota Children’s Clinic, 2-6-18 Katsuyamakita Ikunoku, Osaka 544-0033, Japan; 2 Department of Public Health, Osaka City University Graduate School of Medicine, 1-4-3 Asahimachi Abenoku, Osaka 545-0051, Japan; ito.kazuya@med.osaka-cu.ac.jp; 3 Tomimoto Pediatric Clinic, 6-6-20 Minatotakadai, Hachinohe 031-0823, Japan; altair@eagle.ocn.ne.jp; 4 Kanazaki Children’s Clinic, 3323 Dotocho Nakaku, Sakai 599-8234, Japan; mitkanazaki@icloud.com; 5 Tsukiyama Child Care Clinic, 484 Akizuki, Wakayama 640-8322, Japan; tsukiyama@mikazukikai.jp (K.T.); akioyuki0628@yahoo.co.jp (A.K.); 6 Sotobo Children’s Clinic, 1880-4 Izumi Misakicho, Isumi 299-4503, Japan; sotobo-child@healthcarenet.jp; 7 Department of Microbiology, Kindai University Faculty of Medicine, 377-2 Ohnohigashi, Osakasayama 589-8511, Japan; mfujita47@med.kindai.ac.jp; 8 KidZ Health Castle, UZ Brussel, Vrije Universiteit Brussel, 1090 Brussels, Belgium; Yvan.Vandenplas@uzbrussel.be

**Keywords:** constipation, *Dialister*, functional gastrointestinal disorder, *Lactobacillus*, magnesium oxide, microbiome, Bristol stool scale

## Abstract

Objective: Chronic functional constipation is a frequent condition. The aim of the study was to evaluate the efficacy of the probiotic *Lactobacillus (L.) reuteri* DSM 17938 and magnesium oxide (MgO) for relieving chronic functional constipation in children. Study design: A prospective, double-blind, placebo-controlled, randomized, and parallel-group trial was conducted in five pediatric outpatient clinics in Japan. Sixty patients who were more than six months old and under six years of age with a diagnosis of functional constipation according to Rome IV criteria were randomly divided into three groups: group A (*n* = 20) received *L. reuteri* DSM 17938 and lactose hydrate as a placebo of MgO; group B (*n* = 19) received *L. reuteri* DSM 17938 and MgO; and group C (*n* = 21) received a placebo of *L. reuteri* DSM 17938 and MgO. Results: All three groups exhibited significant improvement in defecation frequency in the fourth week compared with the baseline condition (group A: *p* < 0.05; group B: *p <* 0.05; group C: *p <* 0.05). The MgO group and combination group showed a significant decrease in stool consistency, but the *L. reuteri* DSM 17938 group did not (group A: *p* = 0.079; group B: *p* < 0.05; group C: *p* < 0.05). MgO significantly suppressed the presence of the genus *Dialister*. Defecation frequency negatively correlated with the frequency of *Clostridiales*-belonging bacteria among the gut microbiome. Conclusions: *L. rueteri* DSM 17938 and MgO were both effective in the management of functional constipation in young children. MgO caused an imbalance in the gastrointestinal microbiome, which was not the case in the probiotic group.

## 1. Introduction

Functional constipation is one of the most common gastrointestinal disorders in childhood. The worldwide prevalence of symptoms of constipation in children varies, with an estimated range of 0.3%−28% [1]. However, the treatments are mainly based on empiricism, and little evidence is available concerning the long-term efficacy. The pathophysiology of functional constipation is multi-factorial and is poorly understood at present. However, an important cause of constipation arises from intrinsic defects in the colonic function during the process of defecation [2].

The World Health Organization defines probiotics as “live microorganisms, which when administered in adequate amounts confer a health benefit on the host” [3]. Probiotics have long been generally considered to provide benefits in functional constipation, although few randomized clinical trials have evaluated the effects of probiotics, and data that support the role of probiotics, especially in children with functional chronic constipation, are limited and contradictory [4]. Furthermore, the evidence concerning comparisons between probiotics and placebo or other laxative medications is scarce and of poor quality [5,6]. 

Recently, studies on the human intestinal microbiome based on a metagenomics approach using next-generation sequencing have been dramatically progressing [7]. However, studies evaluating the relationship between changes in clinical symptoms of functional constipation and the intestinal microbiome are lacking. Molecular approaches based on the 16S rDNA gene sequence instead of culture-dependent technology are able to provide detailed classification and diversity information for taxonomy of microbes. Dysbiosis of the gut microbiota may contribute to constipation. Therefore, prebiotics, probiotics, synbiotics, and even fecal microbiota transplantation (FMT) were studied [8]. However, because the types of probiotics were various and the number of patients were small, it is difficult to evaluate the efficacy [8]. Additionally, genetic and non-genetic factors, including maternal and pregnancy-related factors, contribute to gut microbiota composition and the development of many conditions, including functional gastrointestinal disorders. 

*Lactobacillus (L.) reuteri* DSM 17938 was suggested as a possible treatment for constipation by increasing defecation frequency [9]. However, more evidence is needed to clarify the relationship between *L. reuteri* DSM 17938, constipation, and defecation frequency. Therefore, the aim of our study was to evaluate the efficacy of the probiotic *L. reuteri* DSM 17938 and the laxative magnesium oxide (MgO) in children with chronic functional constipation. MgO is an osmotic laxative that has been used in Japan as a stool softener for decades [10]. We also analyzed the relationship between the clinical symptoms and the intestinal microbiome before and after treatment. 

## 2. Methods

### 2.1. Patients

A prospective, double-blind, placebo-controlled, randomized, and parallel-group trial was conducted between January and December 2017. Randomization was performed by a permuted block method. Participants, care givers, and physicians were blinded after the assignment. The study was approved by the ethics committee of Wakayama University of Medicine (approval number 1883). Written informed consent was obtained from all parents. 

In order to be eligible, patients had to be more than six months old or under six years of age, with a diagnosis of functional constipation according to the Rome IV criteria (constipation for at least one month and including at least two of the following criteria: two or fewer defecations per week, a history of excessive stool retention, a history of painful or hard bowel movements, a history of large-diameter stools, presence of a large fecal mass in the rectum, and incontinence after the acquisition of toileting skills) [11]. Children were collected from five pediatric outpatient clinics in Japan (clinic A: *n* = 2; B: *n* = 20; C: *n* = 15; D: *n* = 14; E: *n* = 9). Subjects with known organic causes of constipation, such as Hirschsprung’s disease, spina bifida, cow’s milk allergy, and metabolic disease, were excluded. All infants had started weaning. Before patients were included in the study, their baseline condition of constipation was evaluated over a period of two weeks using a defecation diary. 

Sixty patients were randomly divided into three groups according to an automatically generated randomization list: group A (*n* = 20) received *L. rueteri* DSM 17938 and lactose hydrate as a placebo of MgO; group B (*n* = 19) received *L. rueteri* DSM 17938 and MgO and lactose hydrate; and group C (*n* = 21) received a placebo of *L. rueteri* DSM 17938 and MgO and lactose hydrate. 

### 2.2. Study Products

*L. reuteri* DSM 17938 was administered at a dose of 10^8^ colony-forming units in 5 drops of a commercially available oil suspension (BioGaia AB, Stockholm, Sweden) 30 minutes after feeding, twice a day for four weeks. MgO was administered at a dose of 30 mg/kg of body weight per day. This study was double blinded and we used identical matching placebo and probiotic. The bottles of probiotic and placebo were completely identical. Because MgO (KENEI Pharmaceutical Co., Ltd.) is a raw material, we mixed the same amount of lactose hydrate for the actual drug. Lactose hydrate, which is a sweetening agent without any drug effect, was used for the placebo of MgO. Two physicians and two pharmacists examined the taste and texture of MgO and the placebo of MgO, and they confirmed that they were indistinguishable. All products were kept refrigerated during this study. The use of other laxatives, antibiotics, probiotics, fermented dairy products, and yogurt were not allowed a month before inclusion and during the study period, and a glycerin suppository was used only when there was no defecation for more than three days. All caregivers of patients, research staff, and physicians were blinded to which treatment group the patients belonged.

### 2.3. Outcomes

Defecation frequency was assessed with a defecation diary. The diary was evaluated by caregivers to check the defecation status (stool frequency, stool consistency and amount of stool). Stool consistency was scored according to the Bristol stool form scale [12] as type 1–7 (type 1: Separate hard lumps, such as nuts; type 2: sausage-shaped but lumpy; type 3: similar to a sausage or snake but with cracks on its surface; type 4: similar to a sausage or snake, smooth and soft; type 5: soft blobs with clear-cut edges; type 6: fluffy pieces with ragged edges, a mushy stool; type 7: watery, no solid pieces). The patients visited the outpatient clinic two weeks before administration and at zero, two, and four weeks after inclusion in the study. The gut microbiome was examined at the start and the end of the study period.

The primary clinical endpoint was defecation frequency at the fourth week after starting treatment compared with the baseline condition. The secondary clinical endpoint was the change in the stool consistency score at the fourth week compared with the baseline condition. As another secondary endpoint, we investigated changes in the gut microbiome profiles using high-throughput sequencing of the V3–V4 region of the bacterial 16S ribosomal RNA gene before and after treatment. A cross-section analysis of the defecation frequency was performed with the taxonomy data. 

Each physician monitored adverse events, collected data, and completed case report forms. The random allocation sequence and data analyses were performed by another researcher using unconsolidated data obtained from individuals’ personal information.

### 2.4. Sample Size

In order to detect a significant improvement in defecation, we estimated a standardized size effect of 1.1–1.2 with a 2-sided 5% significance level and a power of 90%. The sample size was calculated to be 16–19 patients per group. Therefore, we decided to include 20 patients in each group, anticipating a dropout rate of 10%.

### 2.5. Statistical Analyses

Regarding the baseline characteristics, the age, defecation frequency, and Bristol stool form score were subjected to an F-test with a one-way analysis of variance (ANOVA). A X^2^ test was used for the sex ratio (Table 1). The least mean square value was estimated by the linear mixed model from the change of baseline defecation frequency and the Bristol stool form score. In the *t*-test used to determine the *p*-value, the error was estimated by the linear mixed model. The null hypothesis was subjected to a two-sided test (Table 2 and Table 3). The analyses of clinical data were performed using the SPSS 16.0 software program (SPSS Inc., Chicago, IL, USA).

The gut microbiome profiles of the bacterial 16S ribosomal RNA gene was performed using MiSeq (Illumina, San Diego, CA, USA). The distribution of alpha diversity in the stool microbiome within groups was measured by the Shannon index and an ANOVA with Holm’s post hoc test was conducted to determine statistical significance (Figure 3). The defecation frequency was cross-analyzed with the taxonomy data by Pearson’s product moment correlation to assess statistical significance (Figure 6).

## 3. Results

Sixty-three patients were recruited to this study and randomly assigned to groups. Three were excluded (two for using antibiotics for otitis media and one that developed Kawasaki disease during study period), so a total of 60 patients enrolled. There were no marked differences between the three groups with respect to gender, age, and baseline condition (Table 1). No adverse event related to any treatment was observed in this study. 

**Figure 1:**
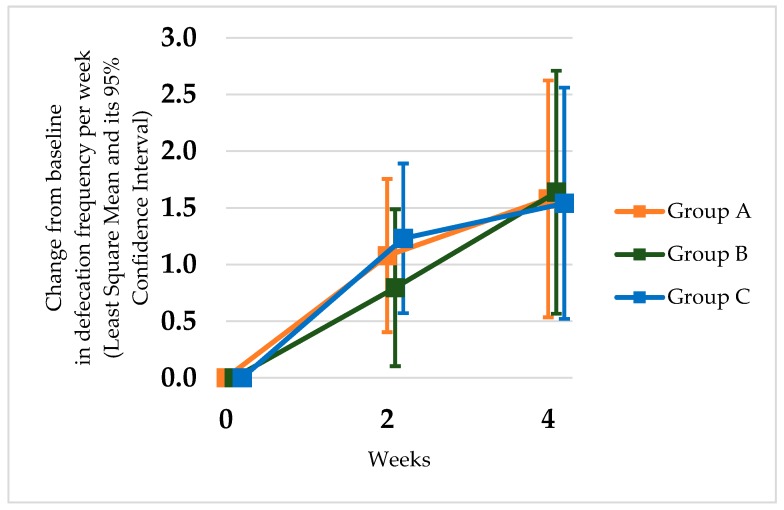
Change from baseline in defecation frequency in the groups administered *Lactobacillus reuteri* DSM 17938 (group A), combination (group B), and magnesium oxide (group C). Time course of the comparison of the differences in the defecation frequency after the initiation of the treatment. All three groups showed a significantly increased defecation frequency at weeks 2 and 4 compared with the baseline.

**Figure 2:**
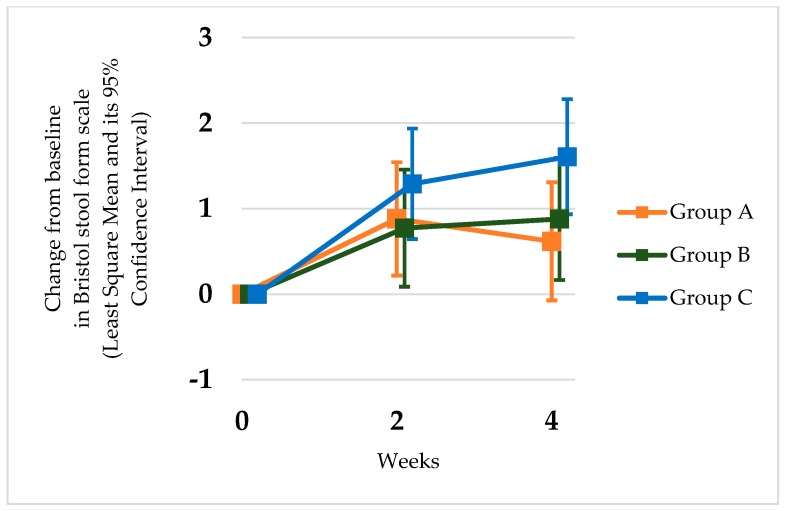
Change from the baseline in the Bristol stool form scale in the groups administered *Lactobacillus reuteri* DSM 17938 (group A), combination (group B), and magnesium oxide (group C). Time course of the comparison of the differences in the Bristol scale at weeks 2 and 4 after the initiation of the treatment. The Bristol stool form scale was increased at weeks 2 and 4 in the combination and magnesium oxide groups compared with the baseline, but the probiotic group did not show any significant changes at week 4.

All three groups exhibited a significant increase in the number of bowel movements during the second and fourth week compared with baseline condition (group A: *p* < 0.05; group B: *p <* 0.05; group C: *p <* 0.05) (Table 2, Figure 1). In each group, the number of defecations had already increased 2 weeks after the start of the study, and the improvement continued up to 4 weeks. In the probiotic and MgO combination treatment group, no synergistic effect was observed compared to a single treatment group regarding defecation frequency. The MgO group and combination group showed a significant decrease in stool consistency, but the *L. reuteri* DSM17938 group did not (group A: *p* = 0.079; group B: *p <* 0.05; group C: *p <* 0.05) (Table 3, Figure 2). The combination therapy of *L. reuteri* and MgO exhibited a significant increase in defecation frequency and decrease in stool consistency. The decrease in stool consistency was more significant in the MgO single therapy group than in the probiotic and MgO combination group. In the end, neither the number of bowel movements or the Bristol stool form scale showed any synergistic effects.

**Figure 3:**
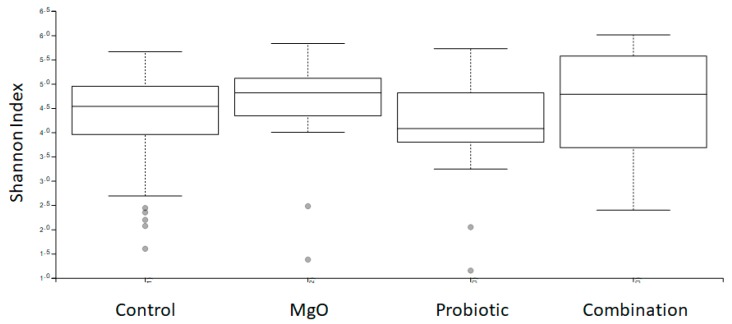
Administration of the probiotic *L. reuteri*, magnesium oxide, or both does not change the distribution of the gut microbiome. Feces were obtained from the patients before and after the treatment. The distribution of alpha diversity within groups was measured by the Shannon index. No marked differences were observed among the groups.

**Figure 4:**
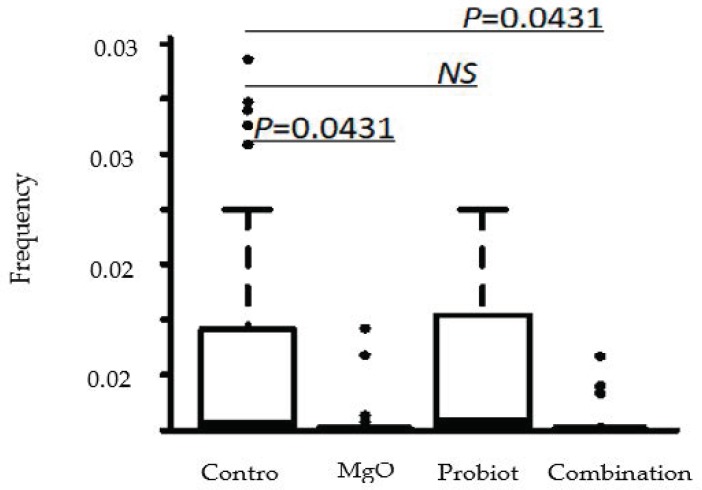
Treatment with magnesium oxide significantly suppressed the presence of the genus *Dialister*.

**Figure 5:**
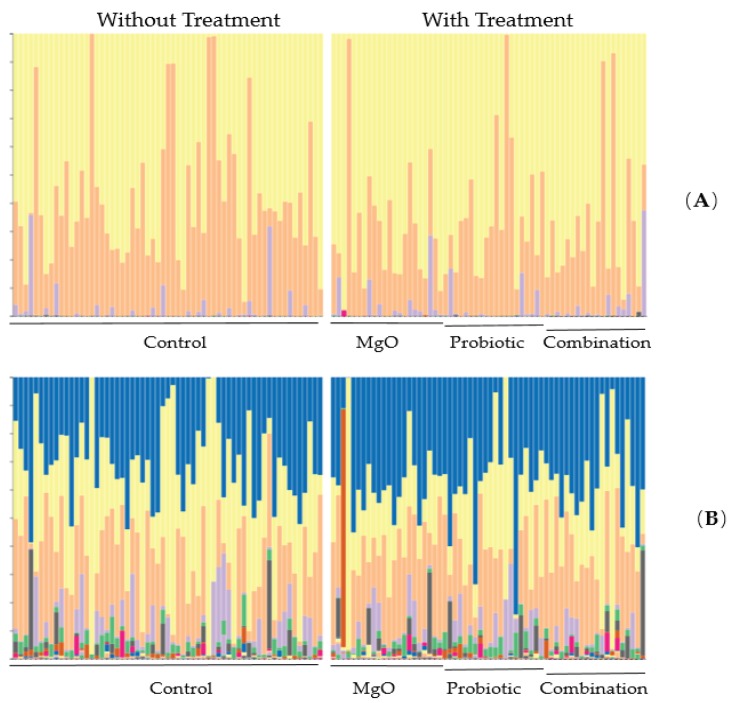
Impact of probiotic *Lactobacillus reuteri* DSM 17938 on gut microbiome in infants with functional chronic constipation. The relative abundance of the gut microbiome was plotted in taxa bars based on the phylum level (**A**) and genus level (**B**).

**Figure 6:**
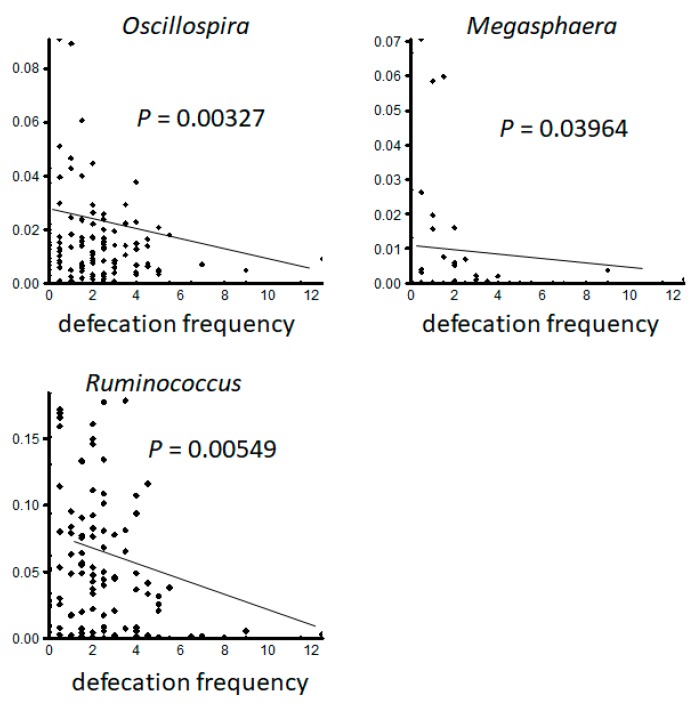
The defecation frequency negatively correlates with the frequency of order *Clostridiales*-belonging bacteria in gut microbiome. The defecation frequency was cross-analyzed with the taxonomy data obtained above and found to correlate negatively with the frequency of some bacteria belonging to the order *Clostridiales*, such as genus *Oscillospira*, *Megasphaera*, and *Ruminococcus*.

Concomitant changes in the gut microbiome were observed. The treatment did not change the alpha distribution of the gut microbiome according to the Shannon index (Figure 3). However, treatment with MgO significantly suppressed the presence of the genus *Dialister* (Figure 4). Furthermore, the defecation frequency negatively correlated with the frequency of order *Clostridiales*-belonging bacteria among the gut microbiome (Figure 5 and Figure 6).

## 4. Discussion

Defecation frequency of constipated children increased significantly in the probiotic and osmotic laxative groups compared with baseline. Furthermore, the consistency of the stool significantly decreased during treatment in the laxative group, although not in the probiotic group. Also in the group with the combination of probiotics and the osmotic laxative, the defecation frequency and stool consistency improved significantly, although not in a synergistic way.

Probiotics are not often used to treat constipation in children, but they are known to induce changes in the composition of the intestinal microbiota, inducing an enhanced intestinal motility, which reduces the transit time [13,14,15]. A meta-analysis showed that probiotics have the potential to increase the stool frequency but exert no significant effect on the stool consistency [16]. These findings are confirmed in our trial. In the present study, *L. reuteri* DSM 17938 was administered twice a day for four weeks, which is twice as frequently as the recommendation. Because of the safety profile of *L. reuteri* DSM 17938, the efficacy of a higher dosages than recommended was tested. Further studies will be needed in order to confirm which dosage and length of treatment are most efficacious.

MgO is an osmotic laxative frequently used in constipation treatment for patients of all ages [10]. Administration of MgO was reported to result in a significant increase of the serum magnesium concentration, but there have been no reports of clinical adverse effects after daily treatment with MgO among children thus far [17]. In children treated longer than one year with MgO at a similar dose (33 mg/kg of body weight/day), serum magnesium levels were 2.4 (2.3–2.5) mg/dL with a normal range of 1.7–2.4 mg/dL, remaining far below the critical toxic value of 4.9 mg/dL. Serum magnesium concentration increased significantly, but not critically, after daily treatment with MgO in constipated children with normal renal function [14]. Symptoms of hypermagnesemia include facial flushing, diarrhea, nausea and vomiting, stomach cramps, lethargy, depression, muscle weakness, hypotension, and heart rhythm disorders. The administration of *Lactobacillus casei rhamnosus* (Lcr) 35 in children with chronic constipation was reported to be effective in increasing the frequency of defecation in a similar trial as ours comparing a probiotic (Lcr 35), MgO (at a higher dose of 50mg/kg of body weight/day), and placebo [18]. There was no statistically significant difference in the efficacy between MgO and Lcr35. Both MgO and Lcr35 decreased the hardness of stool, but details concerning the stool consistency were not described in that report. According to the results from another randomized control trial, Lcr 35 was not more effective than placebo in the management of functional constipation in children [19]. There was a significant increase in the frequency of defecation from baseline in both the placebo group and in the Lcr35 group, but the defecation frequency in the placebo group was significantly greater than that in the probiotic group [19]. Coccorullo et al. reported that infants treated with *L. reuteri* DSM 17938 at the commercially recommended dosage had a significantly higher frequency of bowel movements than a placebo group [9]. However, there was no marked improvement in stool consistency (as in our trial) or episodes of inconsolable crying [9]. A probiotic mixture including *Bifidobacterium breve*, *Bifidobacterium infantis*, and *Bifidobacterium longum* with polyethylene glycol compared to polyethylene glycol alone were equally effective in the treatment of children with chronic constipation [20]. In that study, a positive trend towards a higher rate of clinical remission was observed in children treated with the probiotic mixture than in those treated with polyethylene glycol alone at one month after the end of the trial [20]. 

We postulated that MgO would be effective in improving the stool consistency and that *L. reuteri* DSM17938 would increase the defecation frequency. Our results showed that both had an effect on the defecation frequency, and MgO improved stool consistency. There is evidence that *L. reuteri* DSM 17938 is superior to other interventions for the treatment of infantile colic in breastfed infants [21]. However, there is insufficient evidence in relation to the treatment of constipation. In terms of its effect on motor behavior, Wu et al. showed that *L. reuteri* DSM 17938 decreases the frequency and velocity of jejunal motor contractions and increases the frequency and velocity of colonic motility [13]. It has been also proposed that *L. reuteri* selectively increases the excitability of colonic neurons and enhances the tonic inhibition of colonic contractile activity [22,23]. In contrast, fermentation products appear to enhance the maximal contraction amplitudes via the direct stimulation of smooth muscle cells [24]. Acetic acids of microbe metabolites induce concentration-dependent reflexive smooth muscle contractions. We believe that the probiotic electrophysiological effect on gut motility and direct stimulation of smooth muscle by probiotic metabolites will affect constipation. 

FMT has been considered as a possible new treatment option in patients with slow-transit constipation. A randomized controlled trial compared the effects of conventional treatment (education, behavioral strategies, and oral laxatives) alone with additional FMT [25]. The cure rate was 30% higher in the FMT group compared with conventional treatment in the group of patents with slow-transit constipation [25].

In one study, de Meij et al. detected no disease-specific separation of fecal samples from 76 children diagnosed with functional constipation using principal coordinate analysis (PCoA) and the calculation of diversity indices [26]. Furthermore, there was no statistically significant difference in the Shannon diversity index between the constipated group and the control group, which is in line with the results of the present study. However, ridge regression was able to discriminate functional constipation patients and healthy controls with 82% accuracy. Several species of major phyla (*Firmicutes, Actinobacteria, Fusobacteria, Verrucomicrobia, Bacteriodetes*, and *Proteobacteria*) were detected in the gut for discrimination between children with functional constipation and matched controls [26]. One of these species, *Ruminococcus* spp., showed a decreased abundance in the functional constipation group compared with the control group [26], contrary to our findings. In our study, treatment did not change alpha distribution of the gut microbiome on any level, although we did detect a negative correlation between defecation frequency and *Clostridiales,* including genera such as *Oscillospira*, *Megasphaera*, and *Ruminococcus*. In this study, species groups that were negatively correlated with the number of bowel movements were found, but a relationship with the treatment group was not revealed. The relation between probiotic treatment and its effects on the gut microbiome needs further examination and evaluation. MgO significantly suppressed the presence of the genus *Dialister.* Although the antimicrobial activities of MgO have been reported recently [27], as far as we could find, this is the first report on the relation between MgO and the genus *Dialister.* The fact that no further synergistic effect was observed in the probiotic and MgO combination group is presumed to be due to the antimicrobial action of MgO. We believe that the effects of MgO on microorganisms deserves further investigation. 

The possibility that the combination therapy did not show any additional effect may be related to the baseline characteristics of group B. We adopted a permuted block method for randomization, but group B tended to be slightly older in age and higher in Bristol stool form scale than other groups. We believe that the possible bias is very slight, but we estimate that more subjects are needed to eliminate this kind of bias.

Constipation is generally attributed to multiple factors. Diet, lifestyle habits, genetics, exercise, and other factors are involved in a complex way and influence defecation frequency and composition; furthermore, all of these factors profoundly influence the human microbiota. In this study, we did not asses the habits of diet, water consumption, or exercise. Further studies are needed on these aspects. Additionally, other symptoms related to constipation, such as abdominal pain, withholding behavior, and fecal soiling, were not collected in this study. These symptoms also need to be assed in further trials. Combination therapy of a probiotic and laxative did not demonstrate any additive effect in the present study. Serum magnesium levels increase with MgO administration, however, administration of probiotics induces changes of the microbiome. Further studies with larger populations will be needed in order to further evaluate the role of probiotics in the management of chronic constipation.

## 5. Conclusions

In our study, we noted a significant improvement in the defecation frequency with *L. reuteri* DSM 17938 in functional constipation. Although evidence is currently insufficient to support a general recommendation concerning the use of probiotics to treat functional childhood constipation, this therapeutic option is generally considered safe. MgO administration significantly suppressed the presence of the genus *Dialister*.

The pathophysiology of constipation is multifactorial and has a complex influence on a patient’s microbiome. Further studies are needed in order to obtain more robust evidence concerning the general use of probiotics in constipation and the relevance of the microbiome in constipated children.

## Figures and Tables

**Table 1:** The χ 2 test was used for the sex ratio, while other parameters were evaluated using the F-test with a one-way analysis of variance. Group A: *Lactobacillus reuteri* DSM 17938; group B: combination; group C: magnesium oxide.

Baseline Characteristics of Subjects				
	Group A	Group B	Group C	*p*-Value
*n*	20	19	21	
Male: Female	11:9	10:9	12:9	0.960
Age, Months/Mean (SD)	32.7 (15.9)	40.3 (17.4)	34.2 (15.2)	0.301
Defecation Frequency Per Week/Mean (SD)	1.68 (1.51)	1.71 (1.35)	1.50 (0.82)	0.848
Bristol Stool Form Scale/Mean (SD)	2.4 (1.69)	3.0 (1.57)	2.4 (1.41)	0.390

**Table 2:** Least square mean. The least mean square value is estimated by the linear mixed model, 95% confidence interval (95% CI), and *p*-value. In the t-test, the error is estimated by the linear mixed model. The null hypothesis was subjected to a two-sided test. Group A: *Lactobacillus reuteri* DSM 17938; group B: combination; group C: magnesium oxide.

Change from Baseline in Defecation Frequency Per Week			
Group	Weeks	Least Square Mean (95% CI)	*p*-Value
A	2	1.08 (0.40, 1.75)	0.002
	4	1.58 (0.53, 2.62)	0.004
B	2	0.79 (0.10, 1.49)	0.025
	4	1.64 (0.57, 2.71)	0.003
C	2	1.23 (0.57, 1.89)	0.000
	4	1.54 (0.52, 2.56)	0.004

**Table 3:** Least square mean. The least mean square value is estimated by the linear mixed model, 95% confidence interval (95% CI), and *p*-value. In the t-test, the error is estimated by the linear mixed model. The null hypothesis was subjected to a two-sided test. Group A: *Lactobacillus reuteri* DSM 17938; group B: combination; group C: magnesium oxide.

Change from Baseline in Bristol Stool Form Scale			
Group	Weeks	Least Square Mean (95% CI)	*p*-Value
A	2	0.88 (0.22, 1.54)	0.010
	4	0.62 (−0.07, 1.31)	0.079
B	2	0.77 (0.09, 1.46)	0.028
	4	0.88 (0.17, 1.59)	0.017
C	2	1.29 (0.64, 1.94)	0.000
	4	1.61 (0.93, 2.28)	0.000

## Abbreviations

MgO	Magnesium oxide
*L. reuteri*	*Lactobacillus reuteri* DSM 17938
FMT	Fecal microbiota transplantation
*Lcr*	*Lactobacillus casei rhamnosus*
